# Measuring protein reduction potentials using ^15^N HSQC NMR spectroscopy^†^

**DOI:** 10.1039/c3cc38952a

**Published:** 2013-01-29

**Authors:** Samantha L. Taylor, Harriet Crawley-Snowdon, Jane L. Wagstaff, Michelle L. Rowe, Mark Shepherd, Richard A. Williamson, Mark J. Howard

**Affiliations:** School of Bioscience, University of Kent, Canterbury, Kent, CT2 7NJ, UK

## Abstract

NMR spectroscopy was used to measure reduction potentials of four redox proteins by following multiple ^15^N HSQC protein resonances across a titration series using mixtures of oxidised and reduced glutathione. Results for PDI a, PDI ab and DsbA agree with the literature and our result for ERp18 confirms this protein as an oxidoreductase of comparable or greater reducing strength than PDI a.

In order for proteins to be stable and to conduct their catalytic functions effciently, proteins must be correctly folded and the presence of disulphide bonds can ensure a protein folds correctly. A family of thiol–disulphide oxidoreductase proteins located within the cells catalyse the formation and rearrangement of disulphide bonds. Typically these enzymes contain an active site motif of CXXC and exist in either the oxidised or reduced state.^1^

PDI (protein disulphide isomerase) is located within the endoplasmic reticulum and aids in the folding of disulphide bond-containing proteins *via* the formation (oxidation), rearrangement (isomerisation) and removal (reduction) of non-native disulphide bonds. Human PDI is a 57 kDa protein that contains four thioredoxin-like domains in the order a, b, b′, a′ with an extended linker (x) between the b′ and a′ domains and an acidic 19-residue C-terminal tail (c). The two catalytic a and a′ domains contain the conserved CGHC active site motif. The b and b′ domains, although non-catalytic, are considered essential for full PDI function, with the b domain providing structural stability and the b′ domain containing the primary ligand binding site.^2-4^ This study uses the a and ab domain fragments of human PDI (*ca*. 14 and 27 kDa in size, respectively). To date, the PDI family contains up to 21 members with differing domain architectures.^1^ ERp18, at 18 kDa, is one of the smallest known members of the PDI family with the thioredoxin fold and a CGAC active site.^5,6^ The fourth protein used in this study is *E. coli* DsbA, the archetypal disulphide oxidoreductase in bacteria.^7^

Determining redox potentials allows the identification of a protein’s role *in vivo*; proteins with low redox potentials in the region of −270 mV, such as thioredoxin,^8^ will have reducing properties, while proteins with higher reduction potentials around −122 mV, such as DsbA, will have oxidising properties.^9^ Reduction potentials have typically been determined by monitoring reactions using tryptophan fluorescence,^10^ or alternatively, using HPLC or radiolabelling. These methods operate by separating and quantifying the relative amounts of reduced and oxidised forms of the protein. However this method could only be applied to proteins with one active site. Recently, the reduction potentials of human PDI were elegantly determined using chemical modification and mass spectrometry to show the influence of adjacent active sites on re-oxidation of PDI by the protein Ero1α.^11^ This approach allows the measurement of two active sites simultaneously using a differential alkylation approach followed by mass spectrometry.

Measurement of the reduction potential using ^15^N HSQC has been achieved previously, which allowed monitoring of one active site cysteine in cytoplasmic desulfothioredoxin from *Desulfovibrio vulgaris*^12^ and 1D ^1^H NMR has been used to study reduction potentials in rubredoxin variants.^13^ Here we demonstrate a novel and much improved method with respect to using ^15^N HSQC NMR data. The cooperative mechanism whereby the protein switches between oxidised and reduced states enables a choice of two resonances to be monitored that mirror the conversion from one oxidation state to the other. In addition, all resonances that significantly shift between oxidised and reduced states are measured and each resonance provides a single Hill equation fit and reduction potential. Fitting each resonance to an individual curve fit provides the opportunity to identify and remove systematic and data specific errors to increase the precision and accuracy of the overall average reduction potential determined. Errors can be overlooked if all data is treated as one within an individual Hill plot.

Proof of concept is demonstrated for proteins containing one active site, however, it could easily be expanded to obtain redox potentials of proteins with two or more active sites. This would require NMR assignments to identify resonances from amino acids that report on each active site. Assignment of resonances to individual amino acids is summarised in the ESI.† Conversely, for proteins with a single active site, no residue-specific assignments are necessary, as only changes in each resonance are required to monitor the oxidative status.

^15^N enriched recombinant human PDI ab and ERp18 were expressed in *E. coli* and purified as previously described.^2,6^ PDI a and DsbA were produced using protocols outlined in the ESI.†

To determine the reduction potential of PDI ab, PDI a, ERp18 and DsbA different ratios of [GSH]/[GSSG] were used where the total concentration ([GSH] + [GSSG]) equalled 5 mM. All NMR data was obtained at 298 K using a 14.1 T (600 MHz ^1^H) Bruker Avance III NMR spectrometer equipped with a QCI-F cryoprobe. All NMR samples were 330 μL within a Shigemi NMR tube and contained either 0.065 mM (PDI a) or 0.3 mM (PDI ab, ERp18, DsbA) protein in 20 mM sodium phosphate buffer (pH 7.3) containing 50 mM sodium chloride and 5% (v/v) deuterium oxide. All buffers had nitrogen gas bubbled through them for 30–60 min to ensure the removal of any dissolved oxygen and all proteins were found to reach redox equilibrium within 5 minutes of setting up each condition. All ^15^N HSQC spectra were acquired with 2048 points in the direct F2 dimension (^1^H) and 256 points in the F1 dimension (^15^N). NMR data processing was completed using NMRpipe and example spectra are shown in Fig. 1. NMR backbone assignments have previously been completed for all the proteins^2,6,14^ and were used to identify the peaks that shifted with oxidation status.

Peak heights and volumes were determined using Analysis v2.2^15^ for each oxidised and reduced peak in the spectrum. Peak volumes provided better curve fits for both the oxidised and reduced peaks. The fraction reduced was determined from this data and fitted in Kaleidagraph version 4.1 (Synergy Software) to the Hill equation to determine the equilibrium constant (K_eq_) using eqn (1), where F_red_ is the fraction of reduced protein. Example data fits to the Hill equation are shown in Fig. 2, where millimolar concentrations were used to generate this plot. Therefore, *K*_eq_ is solved from eqn (1) in millimolar units.

(1) $$F_{\text{red}}=({\left[\mathrm{GSH}\right]}^{2}∕[\mathrm{GSSG}])∕(K_{\mathrm{eq}}+{\left[\mathrm{GSH}\right]}^{2}∕[\mathrm{GSSG}])$$

The reduction potential of each protein was then determined by the Nernst equation (eqn (2)) where *T* = 298 K, *n* = 2, and the standard reduction potential of $\mathrm{GSH}\left(E_{0(\mathrm{GSH})}^{\prime }\right)=-240\;\mathrm{mV}$. If *R* is defined as the molar gas constant, *K*_eq_ must be used with molar concentration units in eqn (2).

(2) $$E_{0}^{\prime }=E_{0(\mathrm{GSH})}^{\prime }-(RT∕nF)\ln(K_{\mathrm{eq}})$$

Both equations have been described previously with respect to mass spectrometry reduction potential determination^11^ and are equally valid with our approach.

Errors were determined using the Levenberg–Marquardt *R*-value for the fit of the Hill equation and two controls were run; fully oxidised protein (in 5 mM GSSG) and fully reduced protein (in 5 mM dithiothreitol (DTT)). DTT was used as it is capable of fully reducing catalytic PDI domains. Individual plots are shown in the ESI.† Average reduction potentials and curve fit error values obtained by this method are summarised in Table 1.

This study has measured the reduction potential at one active site of the protein, however, the reduction potentials found in this study are similar to those previously determined.^1,8,11^

The use of multiple resonances to determine the reduction potential is extremely useful. For example, PDI a, which contains 120 amino acids and one CGHC active site, provided ^15^N HSQC spectra with over 18 backbone amide cross peaks with significant chemical shift changes between the reduced and oxidised states. This provides a degree of data redundancy allowing weak or overlapped peaks to be removal from the analysis and a higher precision in the final value of the reduction potential determined. Across all moving resonances in PDI a, individual reduction potentials were calculated and ranged from −148.73 mV to −140.39 mV. Using only optimal data from resonances that were not overlapped or of low signal to noise enables a value of −144.4 ± 0.7 mV to be quoted. We also measured the reduction potential of PDI a and PDI ab as −144.6 ± 1.1 mV and −157.0 ± 2.1 mV respectively *via* single Hill plots that used all resonance data simultaneously; these plot are available in the ESI.† However, our approach provides smaller errors and hence greater precision because handling resonance data separately allows for the identification and exclusion of poor data.

The values for PDI a are consistent with values previously published, where the reduction potential varies from − 110 mV^16^ to −190 mV.^17^ PDI ab data is also consistent and confirms that the addition of the structural b domain does not significantly alter the reduction potential of the catalytic a domain. This data confirms our method is equally applicable for larger proteins as PDI ab is *ca*. 27 kDa. The molecular weight limit for this method will be in excess of 60 kDa when acquiring TROSY optimised ^15^N–^1^H HSQC data. ERp18 provided a reduction potential of −161.5 ± 3.3 mV that suggests this protein is slightly more reducing than PDI a. ERp18 was previous reported to have a similar reduction potential to PDI a, but the value reported was considered of low precision.^5^ The value obtained for DsbA is also within the range of that determined by other approaches.^18,19^

When monitoring the redox changes in a protein, either peak height or volume could be used although peak volumes tended to provide better data fitting to the Hill equation and so was preferred. Our data reveals that reduction potentials for each protein could be determined by measuring peak volumes from only the reduced peak in the spectrum. Equally, the oxidised peak could be used to determine the fraction reduced, but care is required because NMR line broadening of the oxidised state resonance may complicate peak height or volume measurements. The effect of peak line broadening was demonstrated with Hill plots and associated reduction potentials obtained using only oxidised resonances for ERp18 and PDI a (data in ESI†). ERp18 is known to exhibit relaxation line broadening in the oxidised state^6^ and the ESI† data demonstrates these data providing poor Hill plot fits and values even when spectrum sensitivity is not an issue as shown in Fig. 1c and from oxidised and reduced ERp18 spectra displayed in the assignment section of the ESI.† We suggest that to prevent inaccuracy, all peak volumes or heights should be measured from either the oxidised or reduced state and not a mixture of the two forms. However, line broadening effects that alter quantitation could be managed by adjusting peak volumes or heights for relaxation using methods similar to those recently described in the study of metabolites.^20^

Overall, this method of measuring protein reduction potentials using ^15^N HSQC NMR is quick and convenient and has great potential to be utilised with proteins containing multiple active sites. This method can also probe the effect of physical and physiological conditions that influence the mechanistic process of disulphide bond biochemistry through different buffer systems, crowding agents and substrates. Whilst in this study the oxidation and reduction of the proteins were conducted using a [GSH]–[GSSG] mixture, this method could also be applied to alternative systems, for example [NADP]/[NADPH].

## Supplementary Material

arrays

## Figures and Tables

**Fig. 1:**
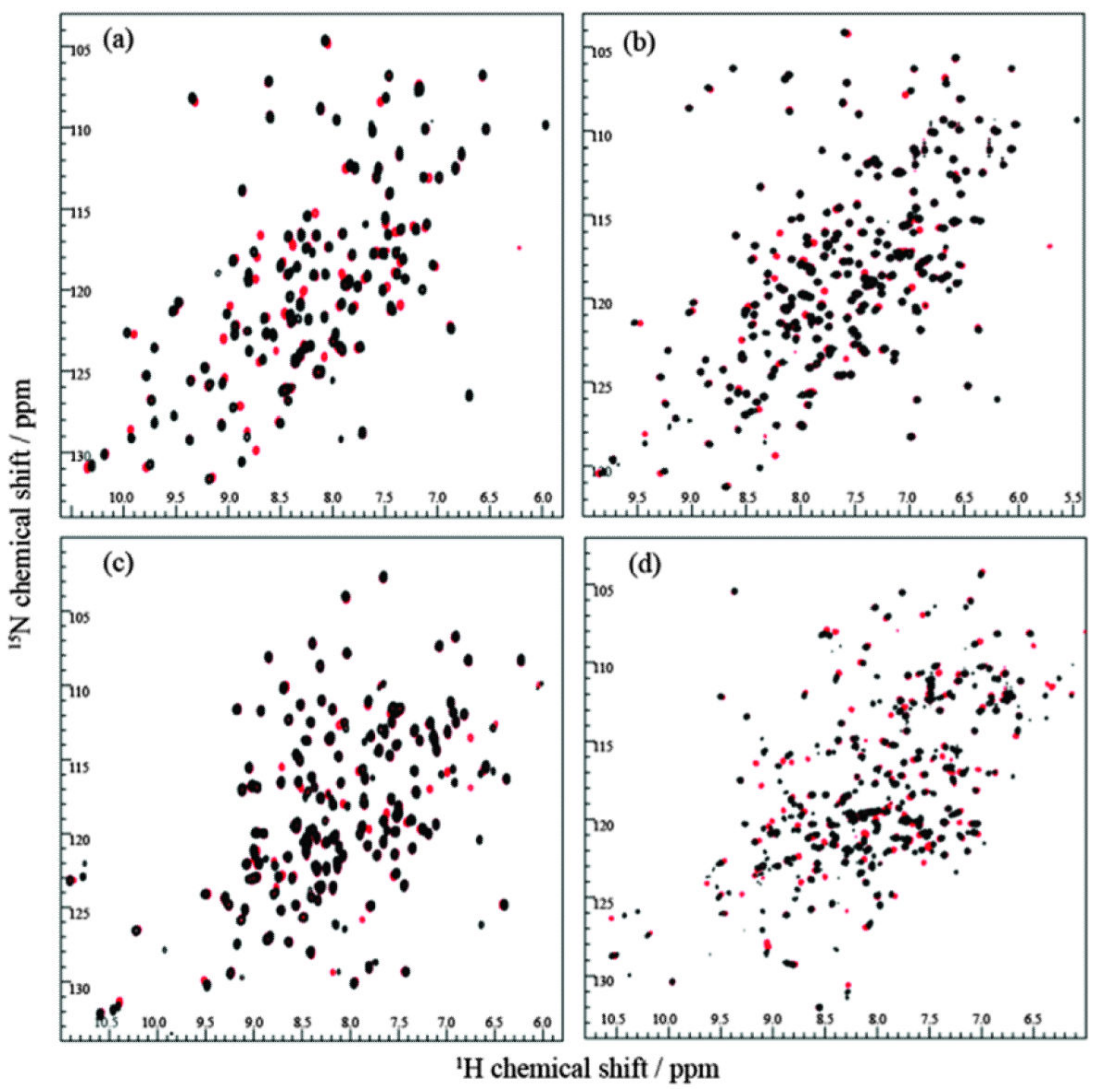
^15^N HSQC spectra of (a) PDI a, (b) PDI ab, (c) ERp18 and (d) DsbA. Fully oxidised spectra are shown in red, and fully reduced spectra are shown in black.

**Fig. 2:**
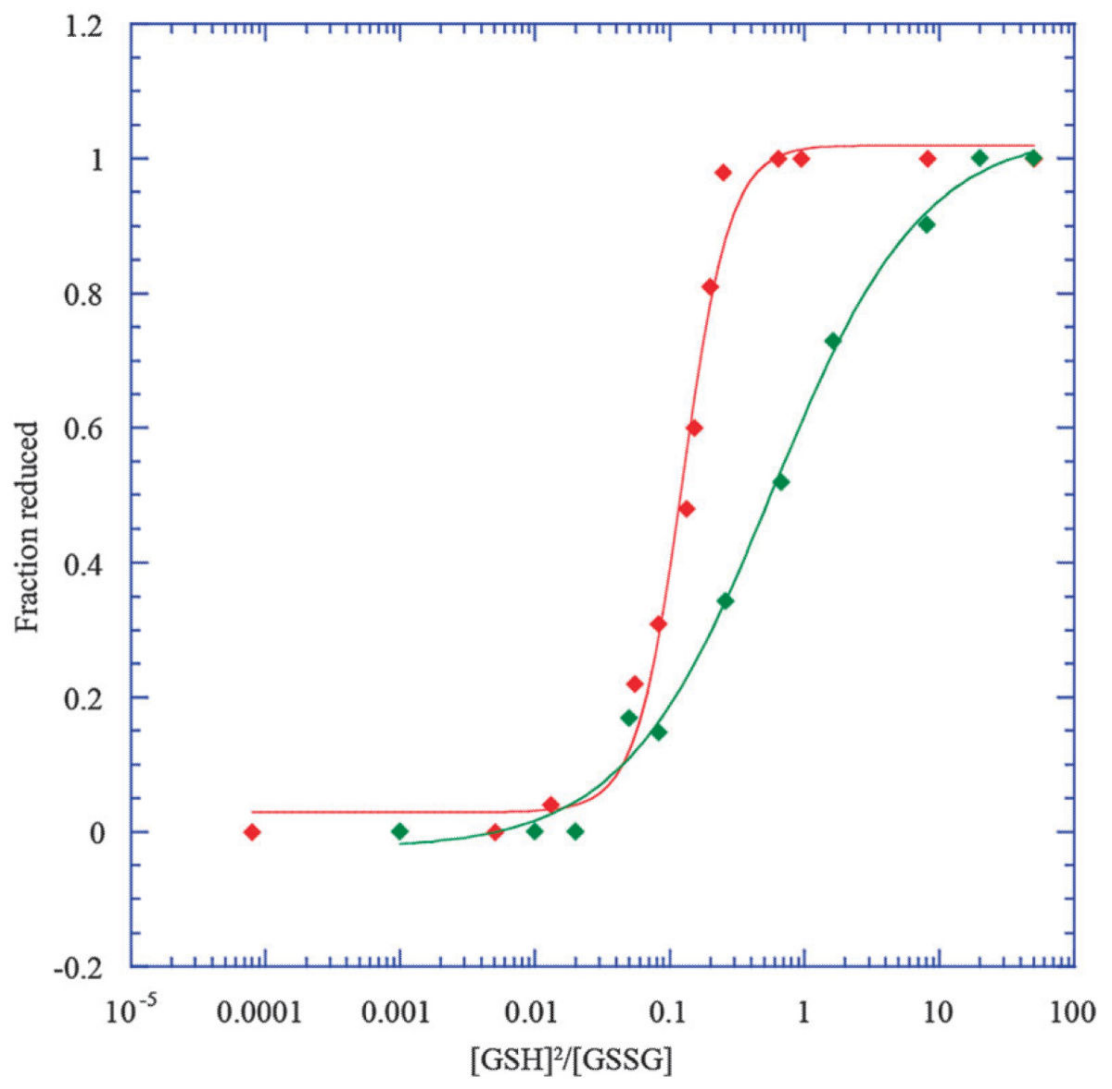
Two fits shown using the Hill equation, PDI a, lysine 14 (green) with a redox potential of −144.43 ± 0.34 mV and DsbA serine 43 (red) with a redox potential of −124.63 ± 0.98 mV.

**Table 1:** *K*_eq_ and mean reduction potential values ($E_{0}^{\prime }$) obtained including number of resonances used in final *K*_eq_ and ($E_{0}^{\prime }$) analysis

	PDI a	PDI ab	ERp18	DsbA
Number of resonances	18	12	6	6
*K*_eq_ (mM)	0.59	1.59	2.61	0.16
$E_{0}^{\prime }(\mathrm{mV})$	−144.4 ± 0.7	−156.8 ± 1.2	−161.5 ± 3.3	−125.6 ± 0.6

## Footnotes

† Electronic supplementary information (ESI) available: Isotopic enrichment notes, protein expression and purification, NMR assignment notes, reduction potential Hill plots for PDI a, ab, ERp18 and DsbA, combined Hill plots of PDI a and ab and oxidised data Hill plots of PDI a and ERp18. See DOI: 10.1039/c3cc38952a
