# Supplementary material for: Measuring protein reduction potentials using 15N HSQC NMR spectroscopy
Source: Chem Commun (Camb). Author manuscript; Available in PMC 2015 Feb 25. (PMC4340554; doi:10.1039/c3cc38952a)
Supplement: arrays [file NIHMS61923-supplement-arrays.pdf]

Electronic Supplementary Material (ESI) for Chemical Communications
This journal is © The Royal Society of Chemistry 2013

Electronic Supplementary Information

Isotopic Enrichment Notes (^{15}N protein production)

Isotopic enrichment of a protein to uniformly ^{15}N label it for ^{15}N HSQC NMR studies involves the recombinant production as outlined below and also outlined in the communication references 2 and 6. Isotopic enrichment is achieved by growing the *E.coli* used in recombinant production in a minimal growth medium (M9 - recipe is generally available[1]) with the addition of a single nitrogen source (typically ^{15}N-ammonium sulphate). This ensures when the protein is recombinantly expressed it is automatically labelled to +90% with ^{15}N. The medium requires a sole carbon source as well and this is usually glucose. If U-^{13}C glucose is used, the protein will become ^{13}C enriched in addition to being ^{15}N enriched.

Expression and Purification of PDI a

PDI **a** was expressed and purified by nickel affinity chromatography as previously described[2]. Purification of the construct by ion exchange chromatography was completed at a higher pH because of the higher pI of the protein construct. Pooled fractions from nickel affinity purification were dialysed overnight in 2 L of 20 mM sodium acetate, pH 8.5. The dialysed protein was then loaded on to 5mL Source 30Q column equilibrated in 20 mM sodium acetate and eluted by a 20 CV gradient with 20mM sodium acetate and 0.5M NaCl pH 8.5.

Expression and Purification of DsbA

DsbA was produced from a pET42b plasmid with a C terminal His-tag. BL21 (DE3) pLyS cells were grown until an A_{600} of 0.6 was reached with shaking at 200 rpm, 37°C; 0.4 mM IPTG was added and the culture induced for 3 h. Cells were sedimented at 6000 rpm for 15 min and then resuspended in binding buffer (25mM Tris, 10 mM imidazole, 0.5 M NaCl, pH 7.0) and frozen at -20°C. Cells were defrosted and sonicated for 4 min (30 s on/ 30 s off) and the mixture centrifuged at 18000 rpm for 15 min. DsbA was purified from the supernatant by nickel affinity and gel exclusion chromatography. The nickel affinity column was washed with binding buffer containing 40 mM imidazole and the DsbA protein eluted with 300 mM imidazole. Fractions containing DsbA were pooled and concentrated and then loaded on to a Superdex G200 column (300 ml bed volume) pre-equilibrated and developed with 20 mM $NaPO_4$ buffer, 50 mM NaCl, pH 7.3.

NMR Assignment of resonances in ^{15}N HSQC

The basis of how NMR HSQC resonances are assigned to particular amino acids is through the process of backbone sequential assignment. This process is well-documented and can be found in a variety of sources.[3-4] The approach typically requires $^{13}C/^{15}N$ enriched protein (see isotopic enrichment above) and a variety of triple resonance experiments described and explained in ESI ref 3-4. ESI Ref 4 is an excellent practical resource for protein NMR for beginners and advanced users. An example of oxidised (A) and reduced (B) ERp18 HSQC assignments that were obtained using triple resonance NMR methods are shown below:

This journal is © The Royal Society of Chemistry 2013

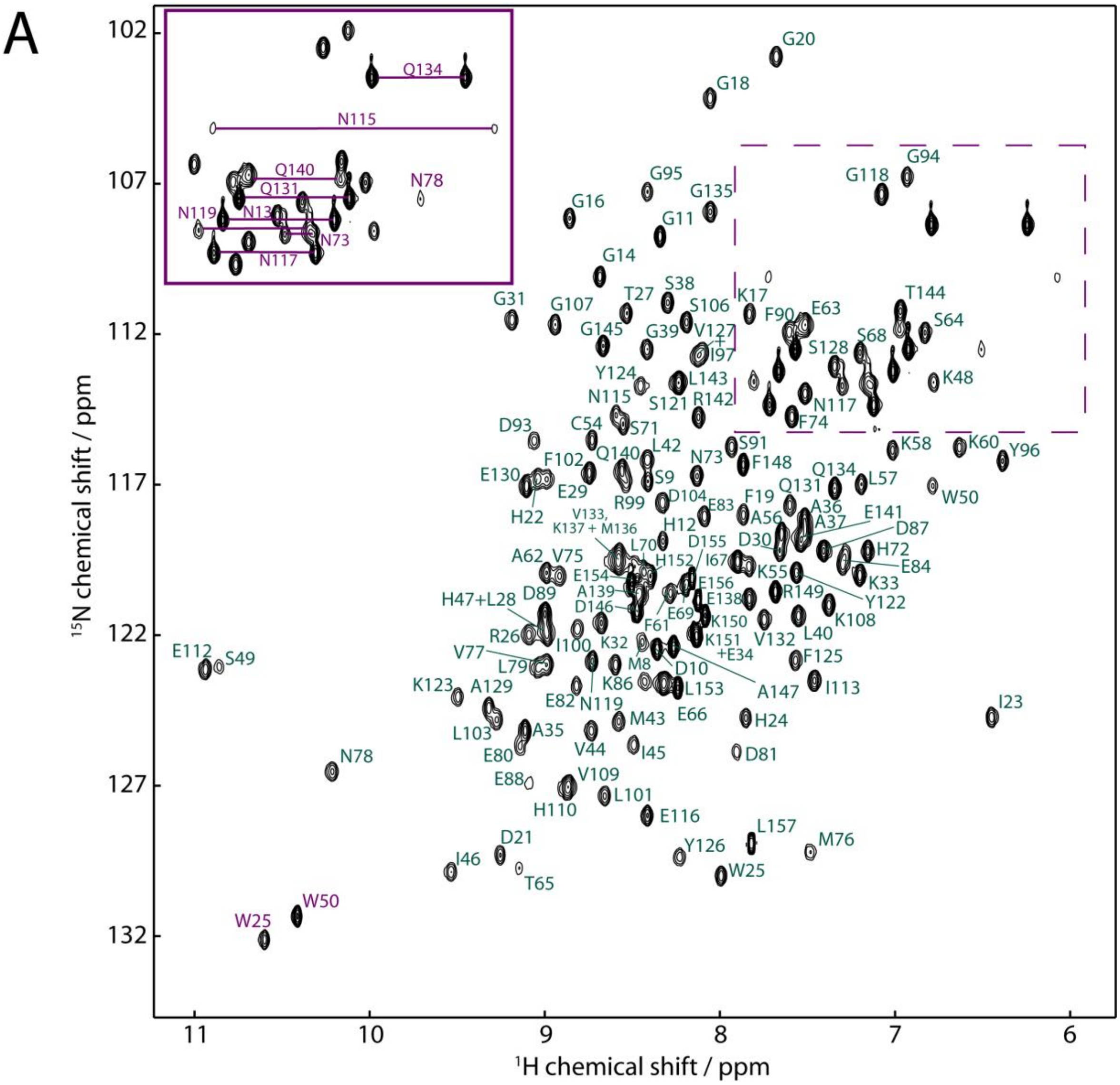

^{15}N-^{1}H HSQC spectra for (A) oxidised ERp18. All known amide resonance assignments are labelled on the spectra. Side chain ^{15}N-^{1}H resonances are shown in purple and main chain resonances are labelled in green. For clarity, the solid box is a copy of the dashed box showing side chain NH resonances only. ^{15}N side chain assignments are discussed in Section 3.3.4.

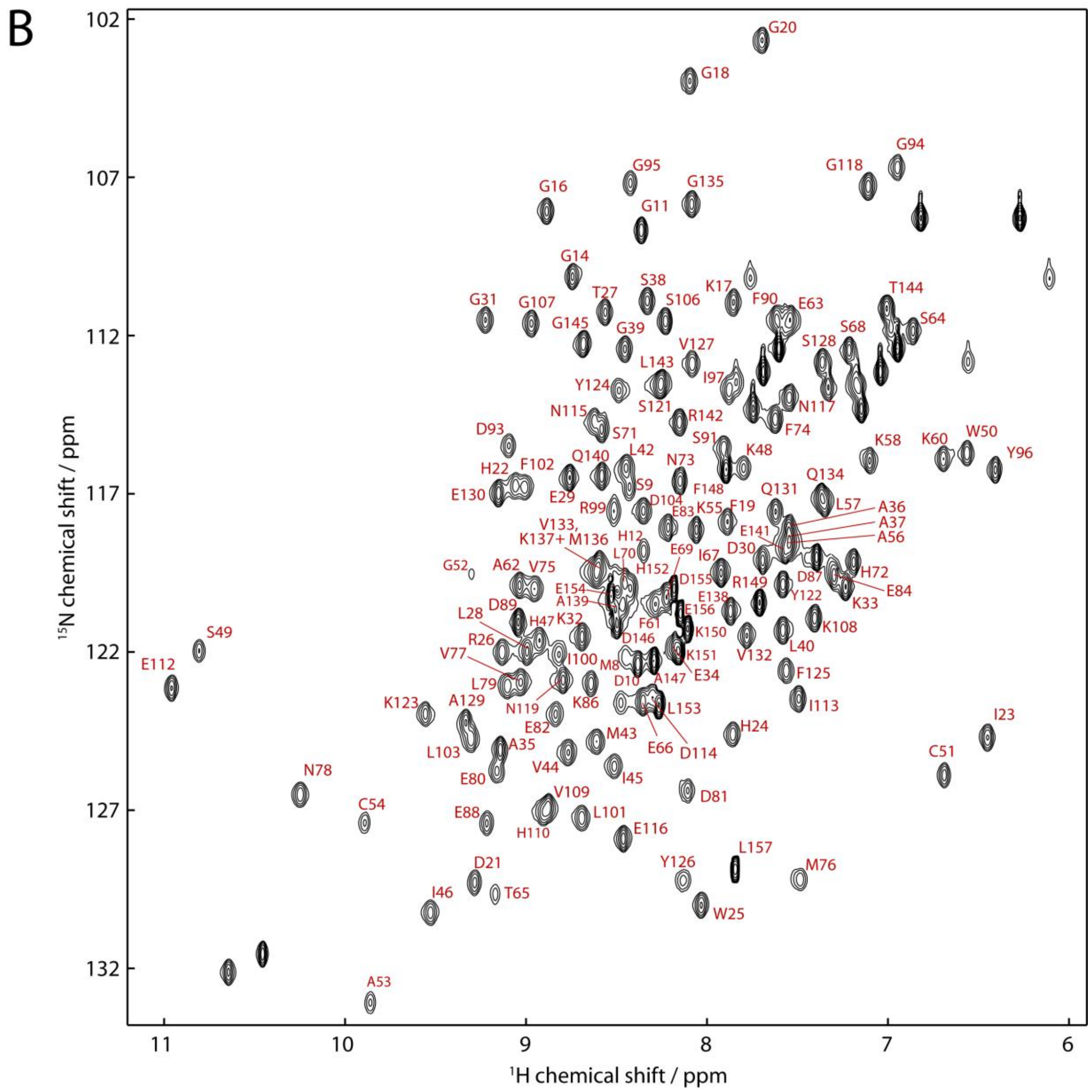

^{15}N-^{1}H HSQC spectra for reduced ERp18. All known amide resonance assignments are labelled on the spectra in red. Side chain resonances have not been labelled as they have not been confirmed by NOESY analysis.

References

[1] J. Sambrook, E.F.Fritsch, E.F and T.Maniatis, Molecular Cloning 2nd Ed., 1989 Vol. 3, p. A.3

[2] L. J. Byrne, A. Sidhu, A. K. Wallis, L. W. Ruddock, R. B. Freedman, M. J. Howard and R. A. Williamson, *Biochem J*, 2009, **432**, 209-217.

[3] J. Cavanagh, W.J.Faitherbrother, A.G.Palmer, M. Rance, N.J. Skelton. Protein NMR Spectroscopy, 2007. Academic Press.

[4] V. Higman, Protein NMR: A Practical Guide. http://www.protein-nmr.org.uk

This journal is © The Royal Society of Chemistry 2013

Reduced PDI a fraction reduced plots

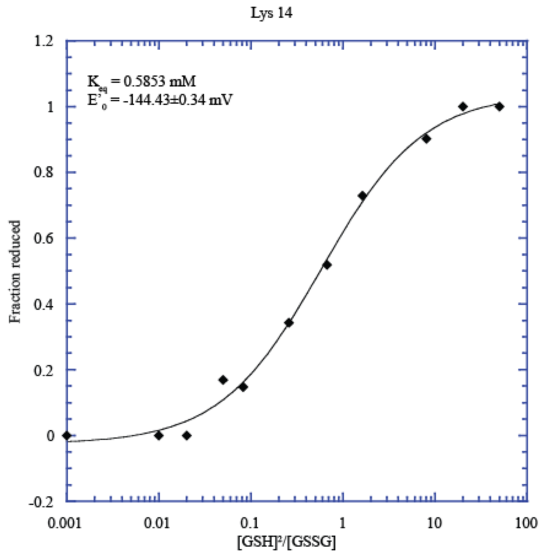

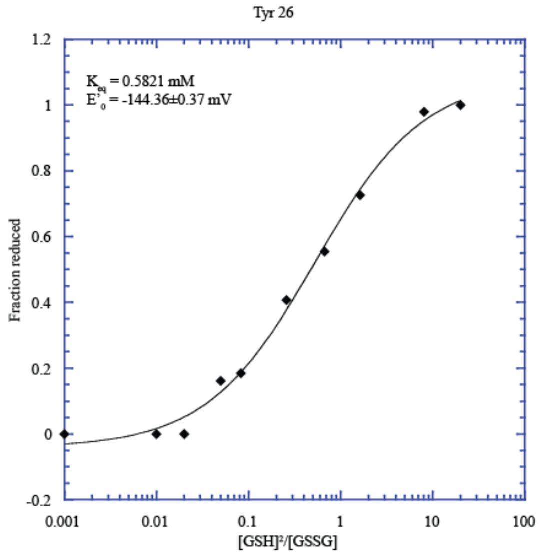

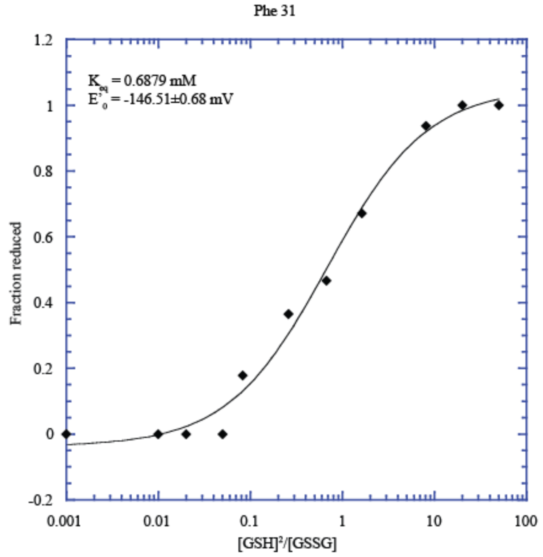

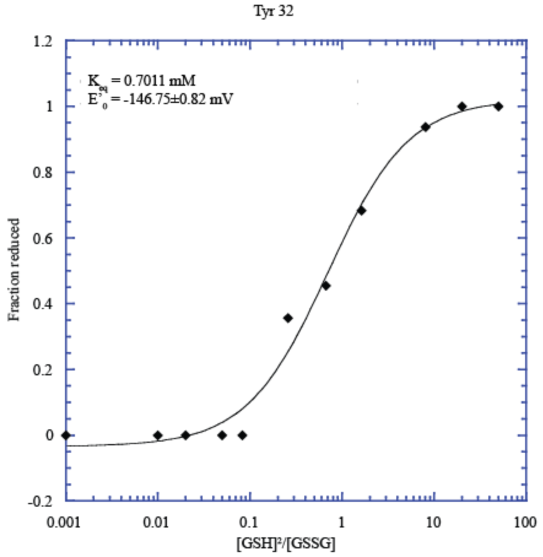

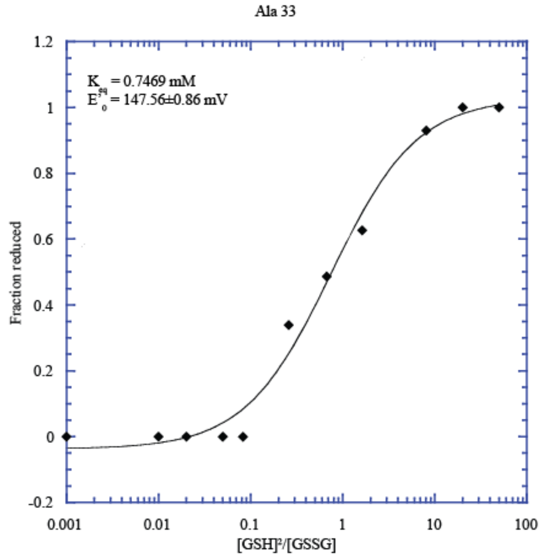

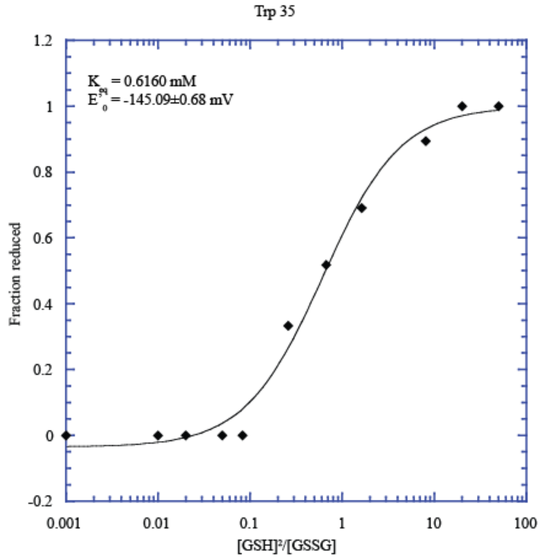

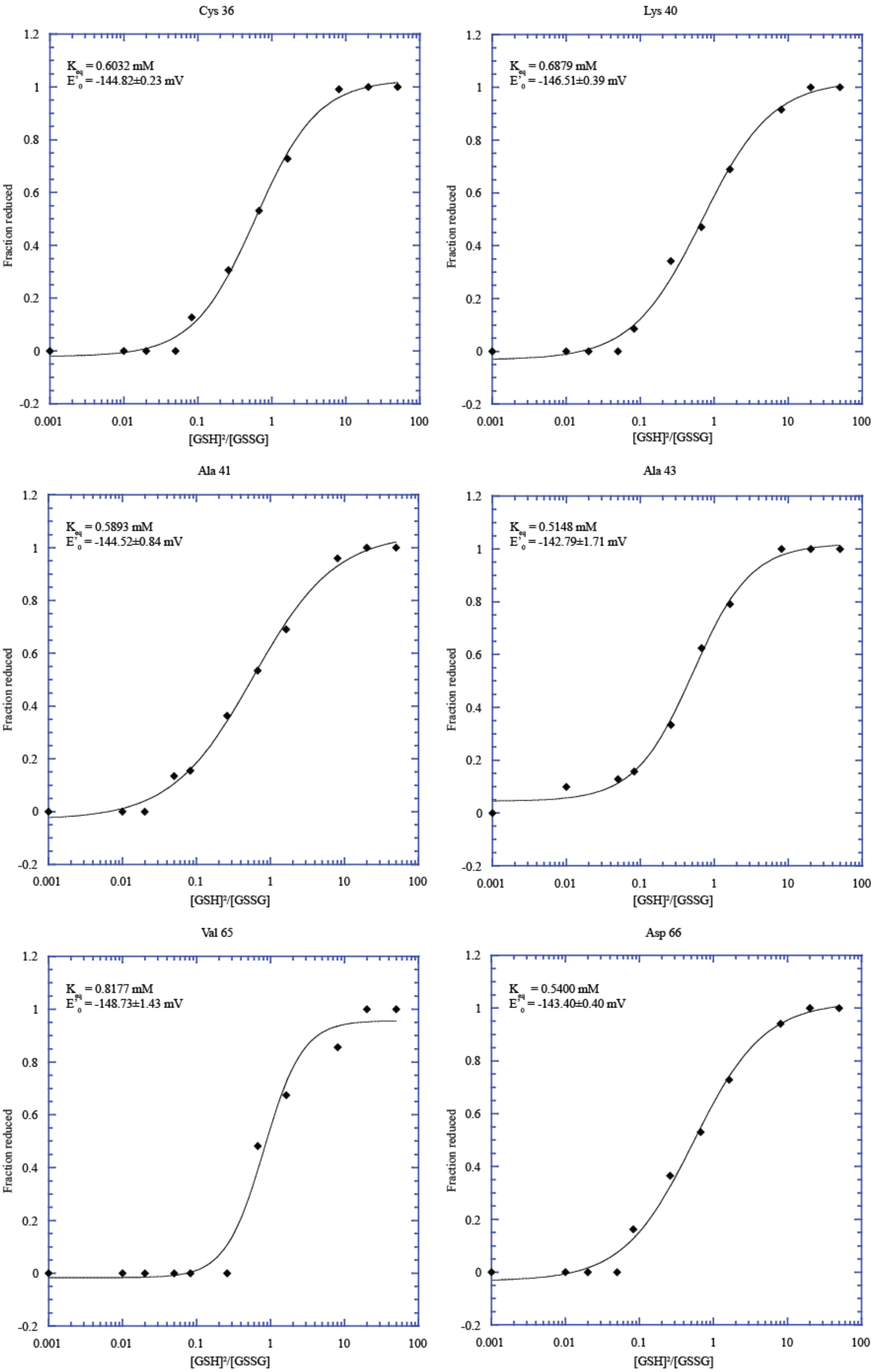
Cys 36
K_eq = 0.6032 mM
E'_0 = -144.82±0.23 mV
Lys 40
K_eq = 0.6879 mM
E'_0 = -146.51±0.39 mV
Ala 41
K_eq = 0.5893 mM
E'_0 = -144.52±0.84 mV
Ala 43
K_eq = 0.5148 mM
E'_0 = -142.79±1.71 mV
Val 65
K_eq = 0.8177 mM
E'_0 = -148.73±1.43 mV
Asp 66
K_eq = 0.5400 mM
E'_0 = -143.40±0.40 mV
Fraction reduced
[GSH]²/[GSSG]
1.2
1
0.8
0.6
0.4
0.2
0
-0.2
0.001
0.01
0.1
1
10
100

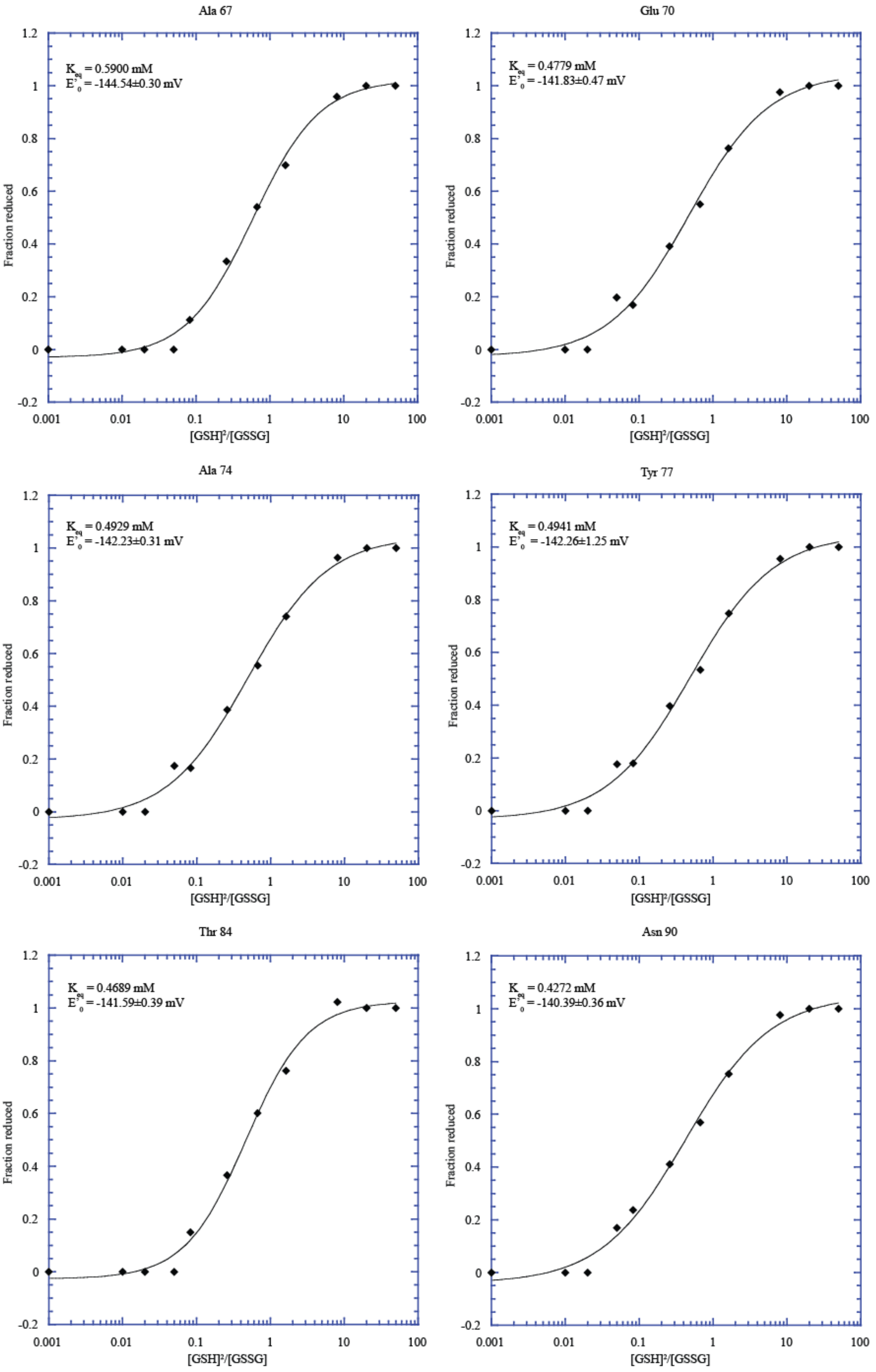
Ala 67
K eq = 0.5900 mM
E°0 = -144.54±0.30 mV
Glu 70
K eq = 0.4779 mM
E°0 = -141.83±0.47 mV
Ala 74
K eq = 0.4929 mM
E°0 = -142.23±0.31 mV
Tyr 77
K eq = 0.4941 mM
E°0 = -142.26±1.25 mV
Thr 84
K eq = 0.4689 mM
E°0 = -141.59±0.39 mV
Asn 90
K eq = 0.4272 mM
E°0 = -140.39±0.36 mV
Fraction reduced
[GSH]²/[GSSG]
1.2
1
0.8
0.6
0.4
0.2
0
-0.2
0.001
0.01
0.1
1
10
100

Electronic Supplementary Material (ESI) for Chemical Communications
This journal is © The Royal Society of Chemistry 2013

Reduced PDI ab fraction reduced plots

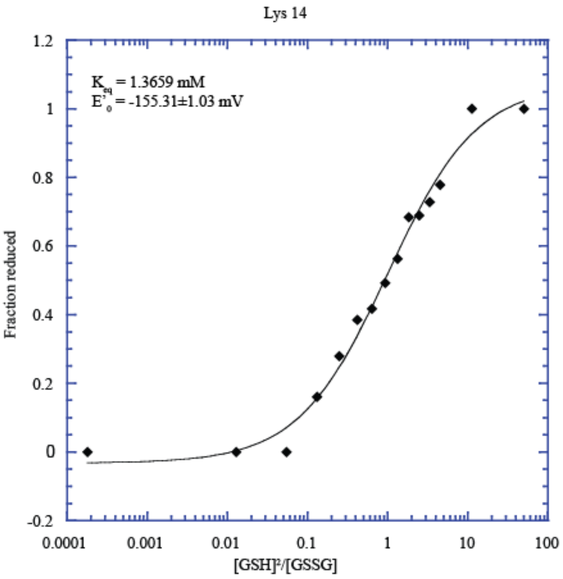

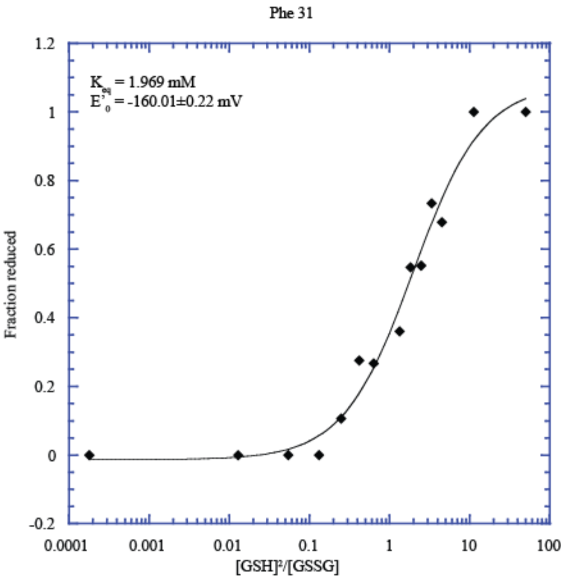

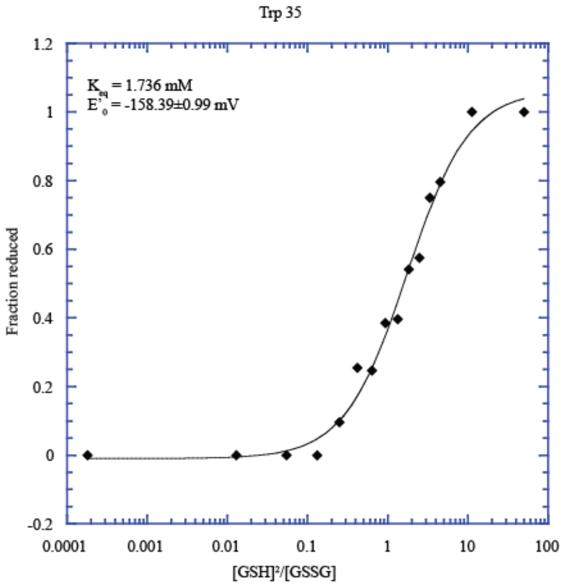

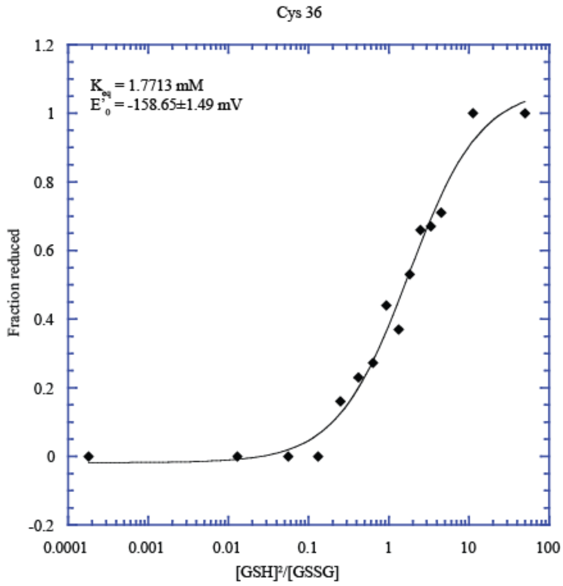

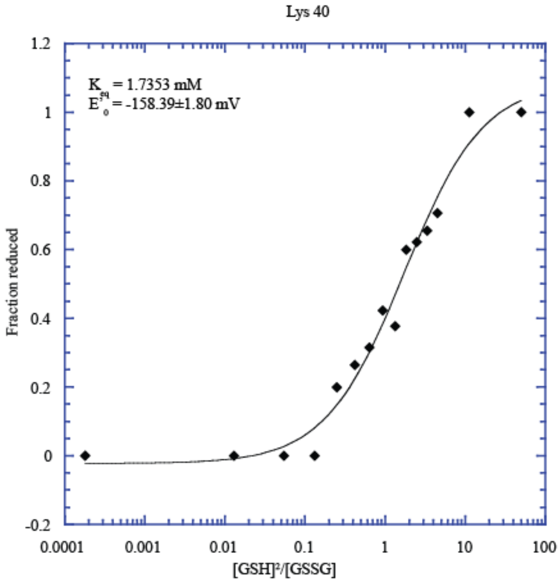

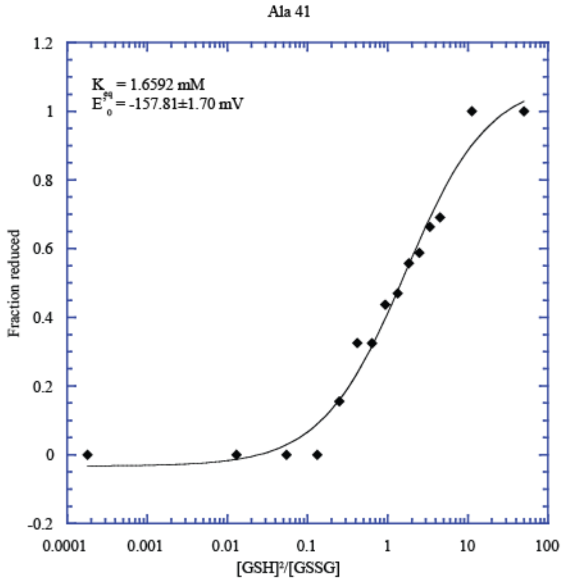

This journal is © The Royal Society of Chemistry 2013

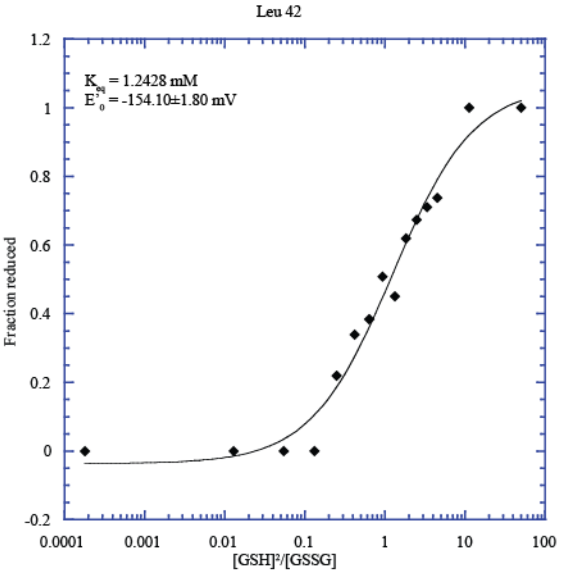

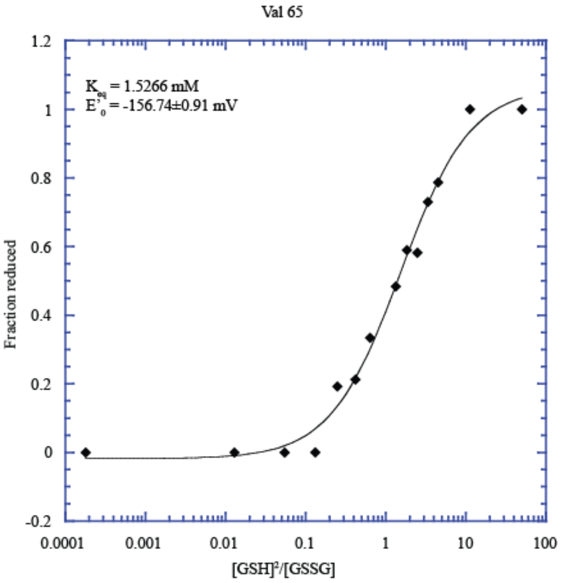

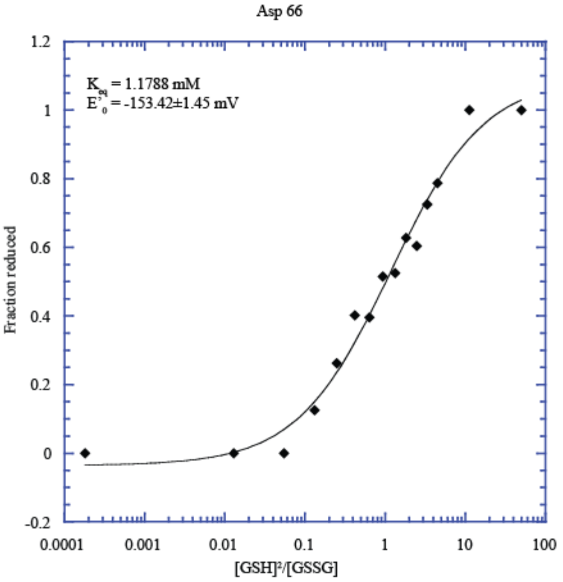

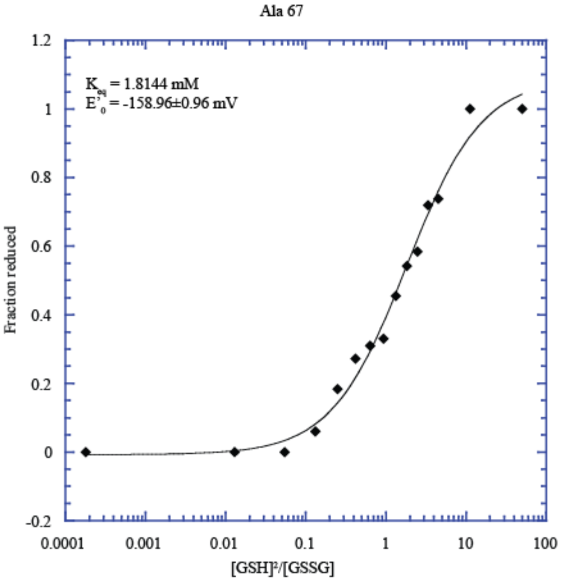

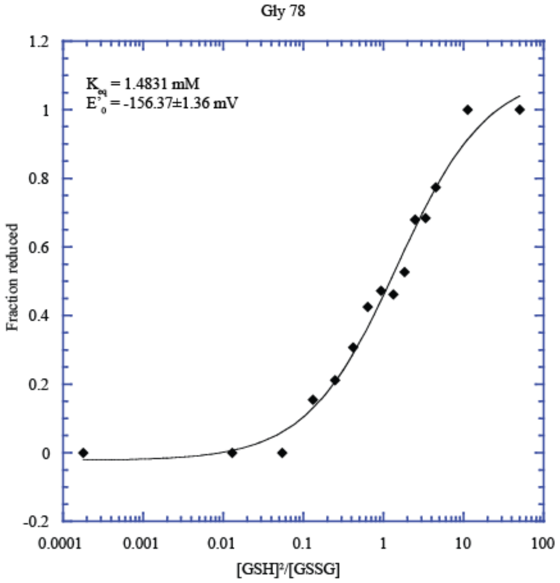

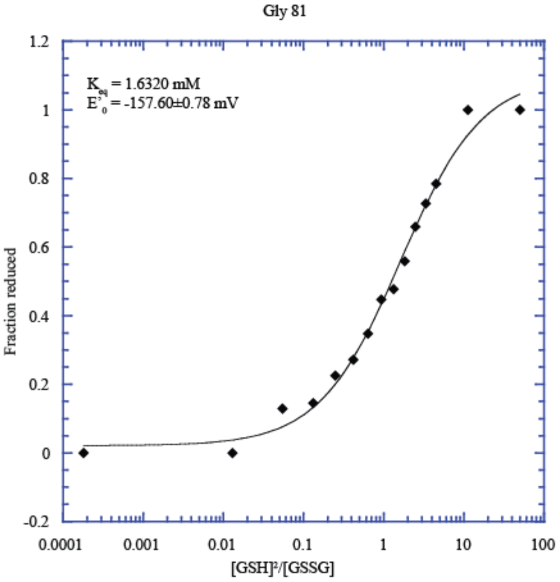

This journal is © The Royal Society of Chemistry 2013

Reduced ERp18 fraction reduced plots

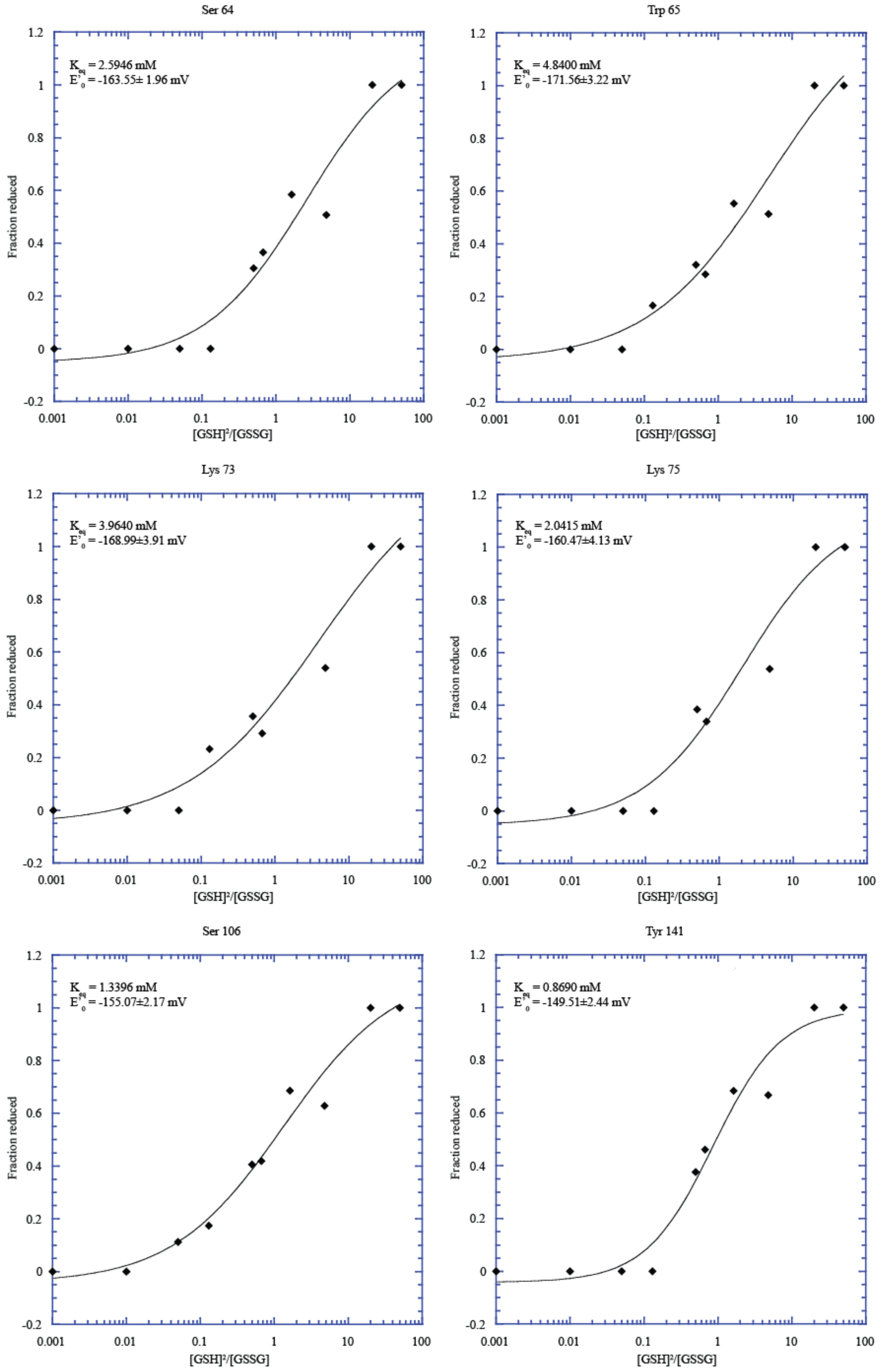

Electronic Supplementary Material (ESI) for Chemical Communications
This journal is © The Royal Society of Chemistry 2013

Reduced DsbA fraction reduced plots

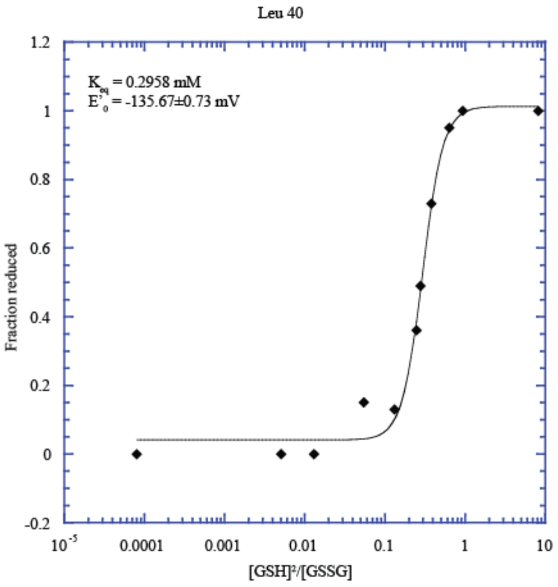

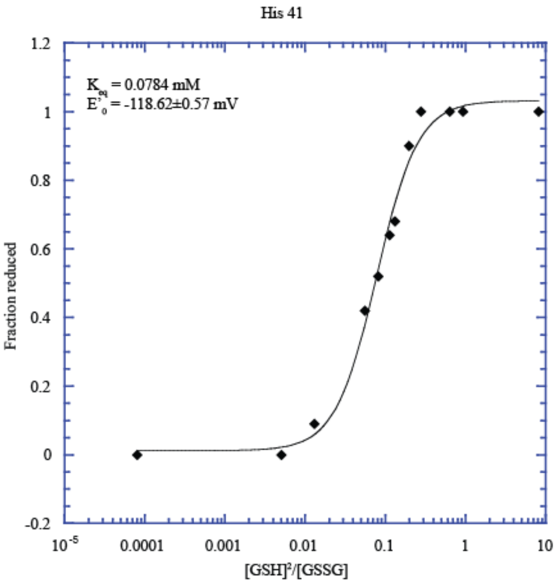

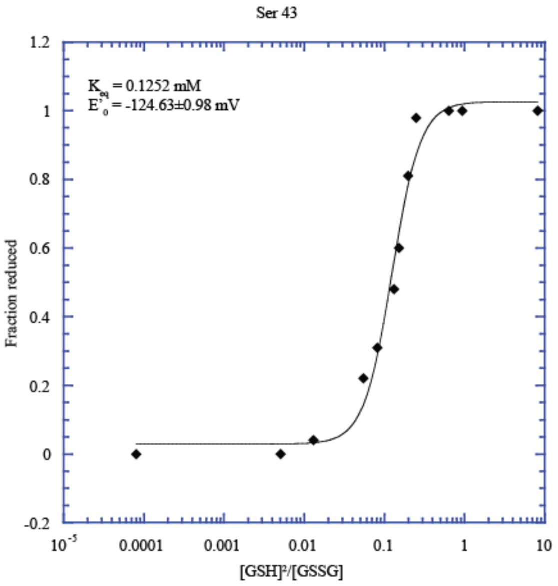

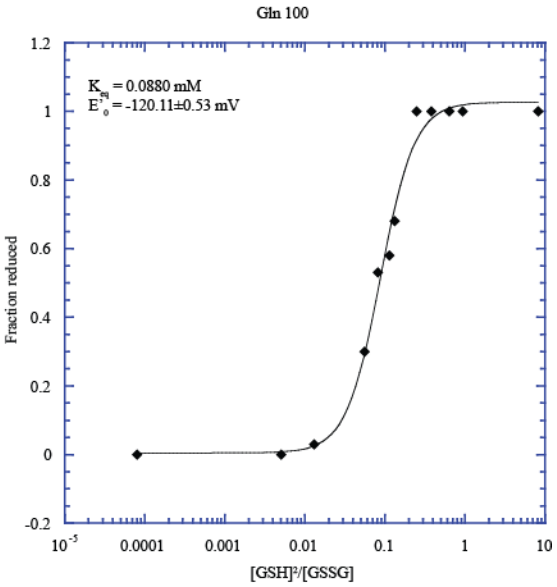

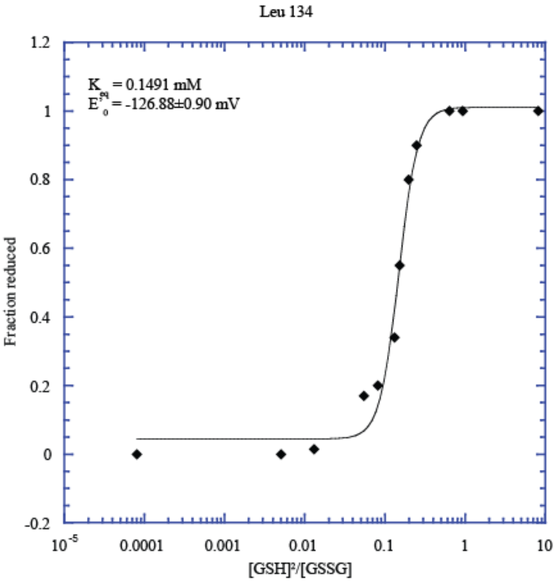

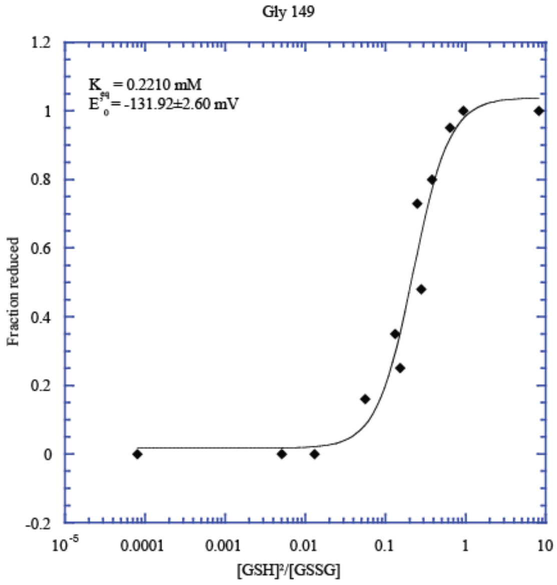

Electronic Supplementary Material (ESI) for Chemical Communications
This journal is © The Royal Society of Chemistry 2013

Reduced PDI a combined data Hill plot

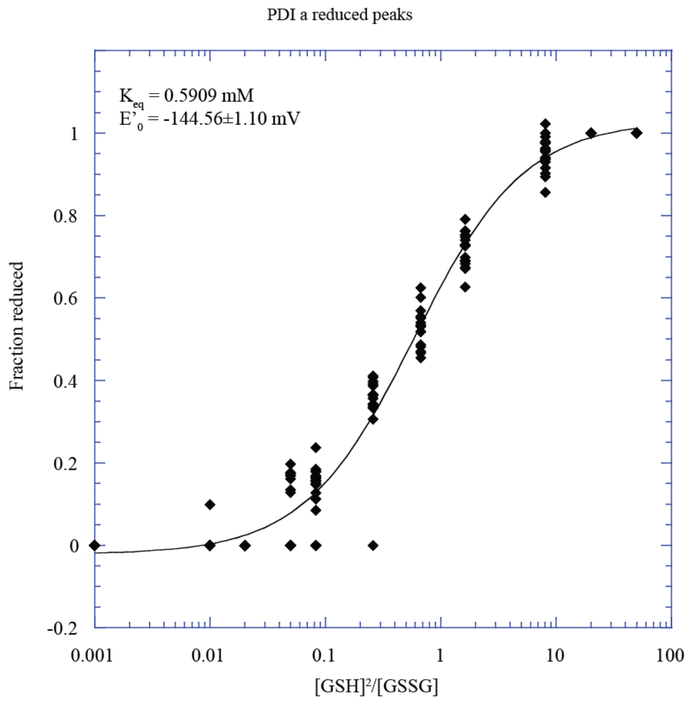

reduced PDI ab combined data Hill plot

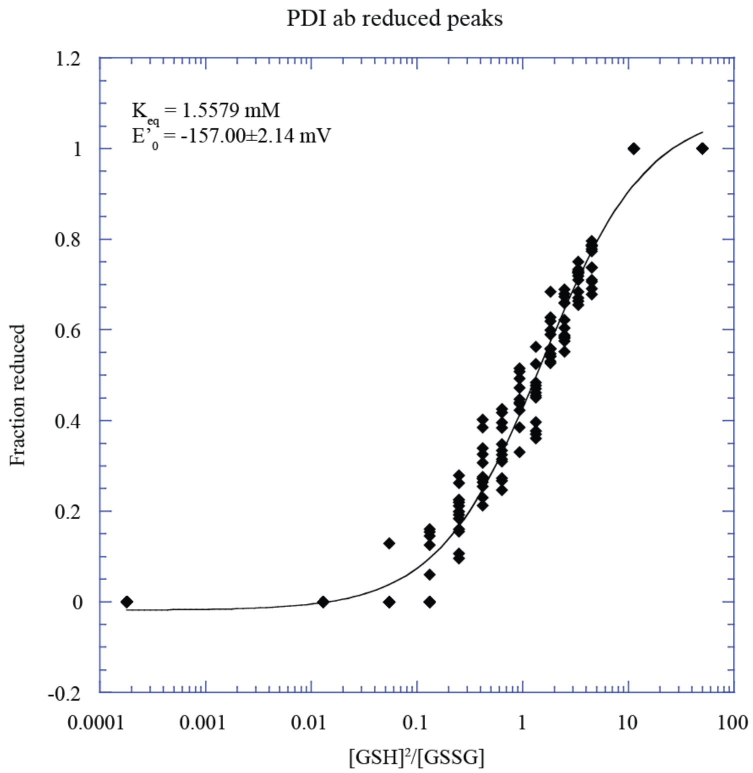

Hill plots using oxidised PDI a resonances

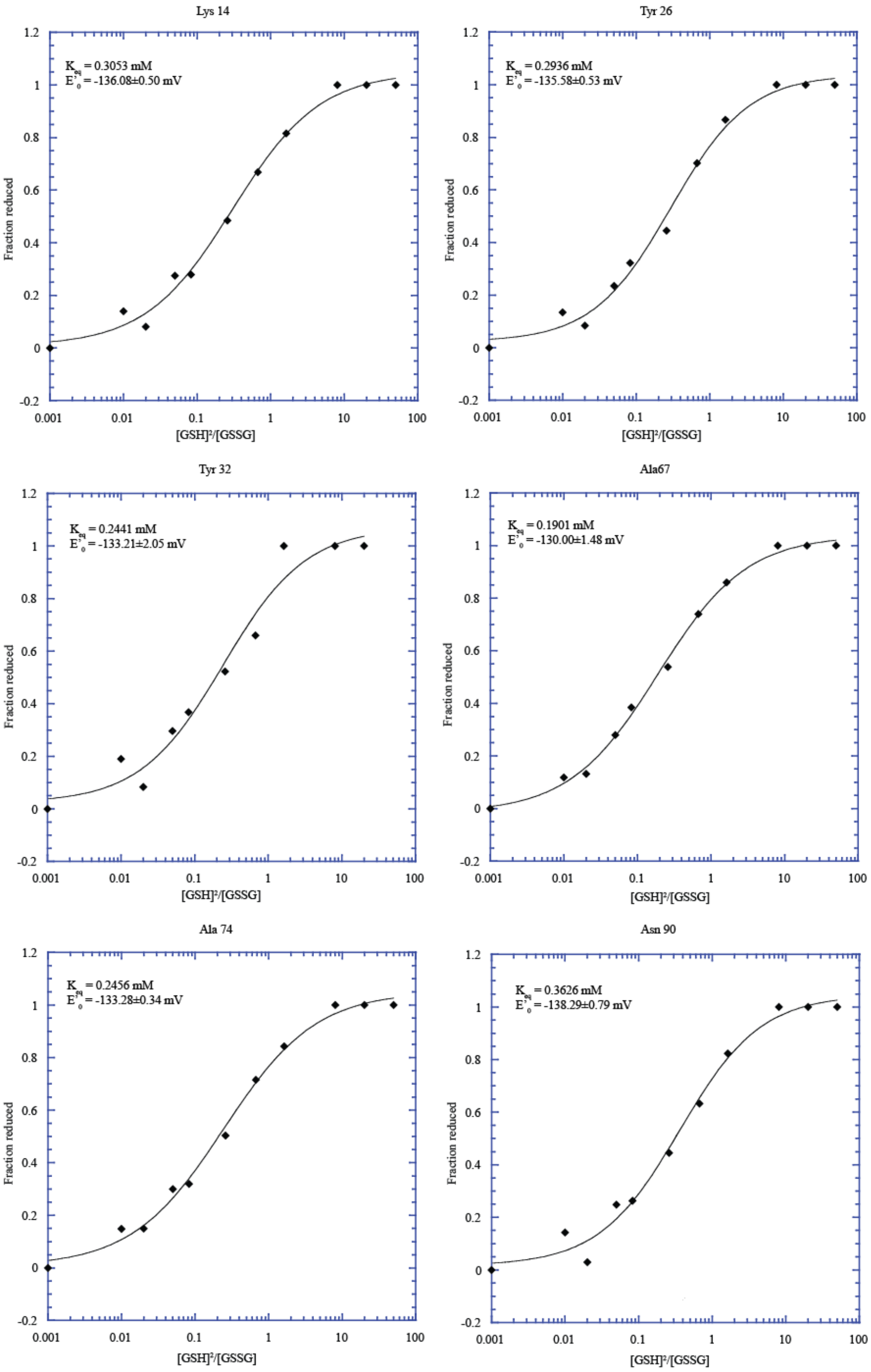

Using oxidised PDI a data above the average E'_o = 134.4± 1.0 mV

This reduction potential is comparable to that reported for PDI **a** (-110 to -190 mV) and that obtained using reduced resonances (144.6 mV).

Hill plots using oxidised ERp18 resonances

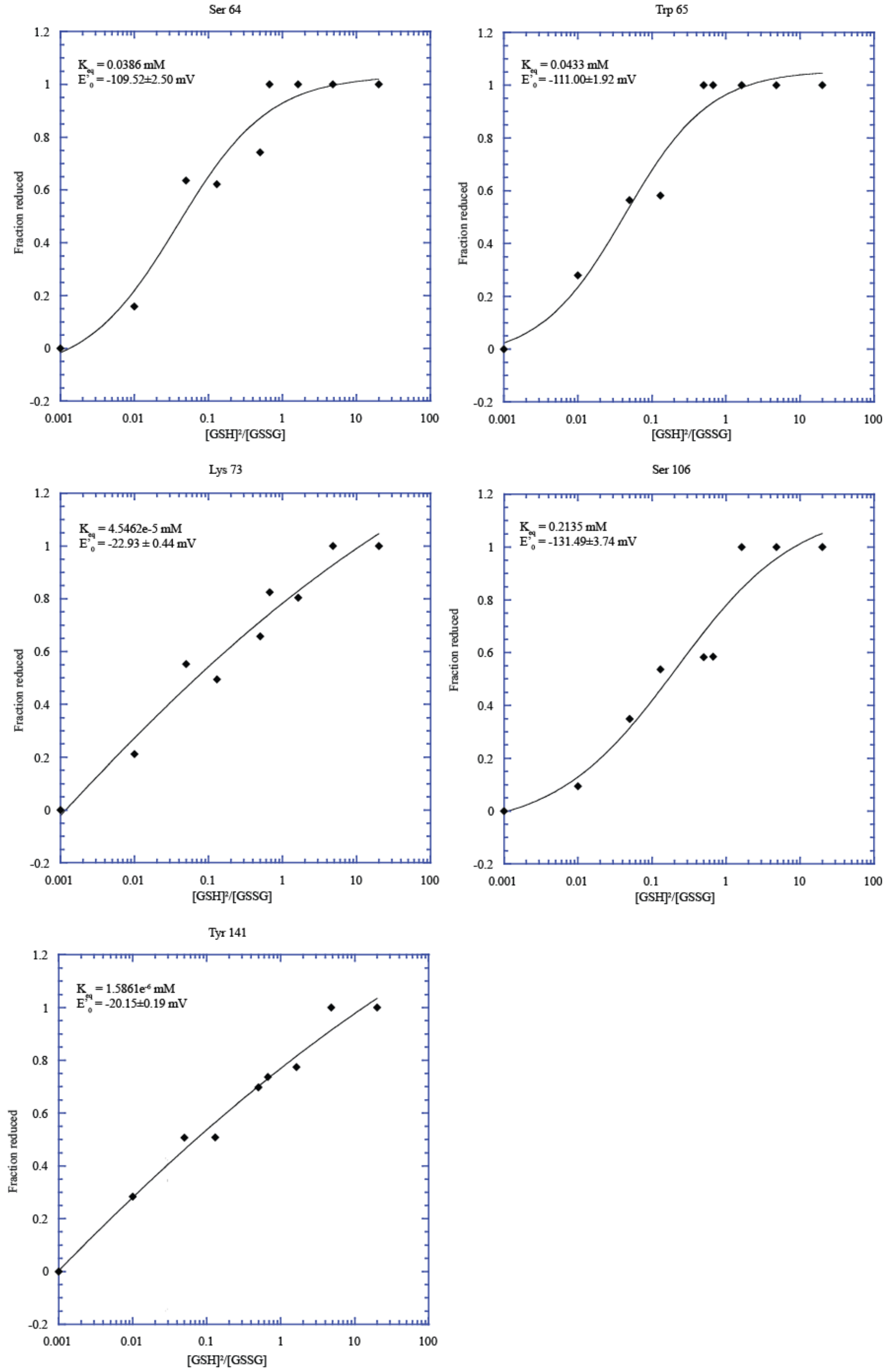

using oxidised ERp18 data above the average E'$_o$ = -79.0 ± 1.8 mV

This reduction potential is poor in comparison to that expected for ERp18 (values similar to PDI **a** -110 to -190 mV). Note that even when such data is not of high quality, the error from the Levenberg-Marquardt R-value can still suggest a reasonable result (see Lys 73 or Tyr 141). Therefore, visual inspection is important in addition to using **<u>separate</u>** Hill plots rather than combining the data into a single Hill plot. A single Hill plot including all points would mask this error. Also, fewer data points could be fitted compared to reduced ERp18 data due to the poorer resolution quality of oxidised ERp18 resonances.
